# Pathways of Glucagon Secretion and Trafficking in the Pancreatic Alpha Cell: Novel Pathways, Proteins, and Targets for Hyperglucagonemia

**DOI:** 10.3389/fendo.2021.726368

**Published:** 2021-09-29

**Authors:** Farzad Asadi, Savita Dhanvantari

**Affiliations:** ^1^ Department of Pathology and Laboratory Medicine, Western University, London, ON, Canada; ^2^ Program in Metabolism and Diabetes, Lawson Health Research Institute, London, ON, Canada; ^3^ Imaging Research Program, Lawson Health Research Institute, London, ON, Canada; ^4^ Department of Medical Biophysics, Western University, London, ON, Canada

**Keywords:** glucagon, secretion, trafficking, alpha cell, hyperglucagonemia, proglucagon processing, secretory granule

## Abstract

Patients with diabetes mellitus exhibit hyperglucagonemia, or excess glucagon secretion, which may be the underlying cause of the hyperglycemia of diabetes. Defective alpha cell secretory responses to glucose and paracrine effectors in both Type 1 and Type 2 diabetes may drive the development of hyperglucagonemia. Therefore, uncovering the mechanisms that regulate glucagon secretion from the pancreatic alpha cell is critical for developing improved treatments for diabetes. In this review, we focus on aspects of alpha cell biology for possible mechanisms for alpha cell dysfunction in diabetes: proglucagon processing, intrinsic and paracrine control of glucagon secretion, secretory granule dynamics, and alterations in intracellular trafficking. We explore possible clues gleaned from these studies in how inhibition of glucagon secretion can be targeted as a treatment for diabetes mellitus.

## Introduction

Glucagon is a 29-amino acid peptide hormone produced by the alpha (α) cells of the pancreatic islet. It is known as the primary glucose counter-regulatory hormone, as its main physiological function is to maintain euglycemia by its actions on the liver to promote glycogenolysis and gluconeogenesis. This glucose counterregulation is impaired in both Type 1 (T1D) and Type 2 (T2D) diabetes (1). Intensive insulin therapy in T1D replaces the beta cell deficiency and corrects fasting and postprandial hyperglycemia, but at increased risks of hypoglycemia (2). These findings suggest that insulin therapy alone may not be adequate for optimal glycemic control. In fact, as stated by the bihormonal hypothesis, diabetes progression may be dependent on both excessive glucagon secretion and insulin deficiency (3), and the glucagonocentric hypothesis ascribes even more importance to alpha cell dysfunction in diabetic hyperglycemia (4). Support for the latter hypothesis seemed to be provided by studies showing that glucagon receptor knockout mice are resistant to the effects of beta cell deficiency induced by streptozotocin treatment (5, 6), providing evidence that blocking glucagon action can provide glycemic control even in the relative absence of insulin. However, more recent studies have shown that blocking the glucagon receptor in absolute insulin deficiency does not prevent hyperglycemia (7, 8), indicating that residual insulin signalling is required in order for glucagon receptor antagonism to be effective.

Nonetheless, the idea that blocking glucagon action could be an additional therapy for diabetes has sparked interest in the development of potential pharmacological interventions that target the hepatic glucagon receptor. Blocking glucagon action can be achieved through: *i)* glucagon receptor antagonists, in particular small molecule antagonists, which can allosterically or competitively inhibit glucagon action (9–11); *ii)* glucagon receptor neutralizing antibodies (12); and *iii)* antisense oligonucleotides against the glucagon receptor (13). However, blockade of the glucagon receptor may induce α-cell hyperplasia and exacerbate hyperaminoacidemia (14–16) through impairments in a liver-alpha cell axis [reviewed in (17)], and increased risk of hyperlipidemia (18). Therefore, long-term use of glucagon receptor blockers may result in harmful metabolic sequelae.

An alternate and possibly safer strategy may be to directly target the intracellular mechanisms that govern the secretion of glucagon from the alpha cell. In this review, we discuss aspects of alpha cell biology that may provide such targets: proglucagon processing, sorting, exocytosis and intracellular trafficking, as well as mechanisms of intrinsic and intra-islet regulation of glucagon secretion.

## Regulated Secretory Pathway of the Pancreatic Alpha Cell

The alpha cell secretory pathway begins with the synthesis of proglucagon in the endoplasmic reticulum. It is then transported through the Golgi to the trans-Golgi network (TGN). Budding immature secretory granules from the TGN contain proglucagon, its processing enzymes and many other proteins (19). But how does proglucagon find its way to the site of granule budding? Based on one hypothesis, proglucagon may contain a sorting signal within its structure that directs it to a specific sorting receptor on the membrane of the TGN.

Investigations into the mechanisms of the sorting of prohormones have revealed the existence of intrinsic sorting signals that can take the form of amphipathic loops, such as for POMC (20) or proinsulin (21), or amphipathic alpha helices, as is the case for the N-terminal region of prosomatostatin, and in the C-terminal regions of PC1/3, PC2, PC5/6a, and CPE (22–24). These signals may interact directly with membrane lipids, in particular with lipid raft regions (25) or with sorting receptors within the TGN, to be sorted into secretory granules. To this end, it has been proposed that membrane-bound form of the processing enzyme carboxypeptidase E (CPE) may be a prohormone sorting receptor (21, 26–28). It was shown that ablation of CPE disrupted the regulated secretion of proopiomelanocortin (POMC), proenkephalin and proinsulin in related cell lines and the CPE^fat^ mouse model, in which CPE is degraded within the pituitary. Additionally, CPE may interact other resident granule proteins, secretogranin III and chromogranin A, to facilitate the sorting of POMC (29) and neuropeptides (30).

Another possible mechanism of prohormone sorting to granules may be simply through selective retention while constitutively secreted proteins are removed from the nascent immature granule. Evidence for this mechanism lies in the fact that the protein composition of immature secretory granules is altered during the process of granule maturation (31). In this context, proinsulin and the enzymes involved in the post-translational processing to mature insulin are retained within the beta cell secretory granule, while other proteins designated for constitutive secretion are removed (31).

By considering all of these findings, it is likely that both receptor-mediated and retention mechanisms operate in the sorting of prohormones into secretory granules. In this scenario, prohormones could be sorted into secretory granules by means of sorting signals, followed by retention within the granule as maturation of secretory granules takes place. The maturation process involves alterations in the components and composition of the secretory granule, by removal of constitutively-secreted proteins, acidification of the granule milieu, and exclusion of water to condense the intragranular environment.

**Figure 1:**
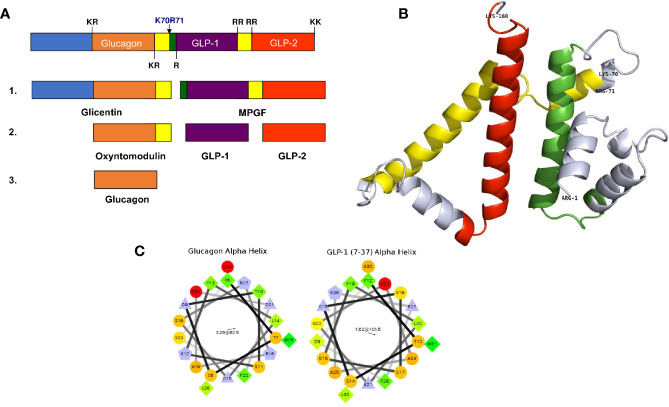
Proglucagon processing and sorting signals. **(A)** A schematic representation of proglucagon showing the major prohormone processing sites that yield the peptides glicentin, oxyntomodulin, glucagon, GLP-1 and GLP-2. **(B)** Computational modelling of the structure of proglucagon showing the alpha helical structures of glucagon (green), GLP-1 (yellow) and GLP-2 (red) (from reference 28) © 2013 Society for Endocrinology. **(C)** Helical wheel projections of the alpha helices contained within glucagon and GLP-1 (7–37) that function as sorting signals to direct proglucagon to the regulated secretory pathway.

The cellular events underlying the sorting of proglucagon to secretory granules have not been fully elucidated, and studying this mechanism is complicated by the multi-step processing of proglucagon. The processing of proglucagon in the alpha cell is largely governed by the prohormone convertase (PC) family of enzymes. Proglucagon processing begins early in the secretory pathway (TGN or immature secretory granule) with cleavage at K_70_R_71_, which yields glicentin and major proglucagon fragment (MPGF) (**Figure 1A**) (32). This site is accessible to a number of processing enzymes, and in the alpha cell, is likely cleaved by furin or PC1/3 in the absence of PC2 (33). Subsequent cleavage of glicentin by PC2 at K_31_R_32_ results in the production of mature glucagon. This cleavage event likely occurs within the mature secretory granule since the enzymatic activity of PC2 is optimal at the acidic pH and millimolar calcium concentrations within secretory granules (33–36). Thus, the sorting of proglucagon into the secretory granule is vital for the generation of active glucagon, and storage within granules assures a robust secretory response in response to physiological need.

Is there evidence for sorting signals and a sorting receptor for proglucagon? Using the alpha cell line αTC1-6, it was shown that siRNA-mediated knockdown of CPE increased constitutive secretion of glucagon; however, the processing of proglucagon to glucagon remained unchanged, indicating that CPE may have an effect on secretion, but not intracellular sorting (28). The search for sorting signals provided more clarity on the mechanisms of proglucagon sorting. Computational modelling of proglucagon indicates a largely disordered structure comprising alpha helices, some of which correspond to glucagon, GLP-1 and GLP-2 (**Figure 1B**). Using Fc-tagged proglucagon-derived peptides that could be detected by immunoprecipitation and immunofluorescence microscopy, it was shown that two dipolar α-helices containing hydrophobic patches with three charged residues within the sequences play roles as sorting signals. One amphipathic α-helix was located within the amino acid sequence of glucagon (SDYSKYLDSRRAQDFVQWLMN), and one within GLP-1 (SDVSSYLEGQAAKEFLAWLVK) (32) (**Figure 1C**). Interestingly, Fc-glicentin was sorted to secretory granules, but Fc-MPGF was not, suggesting that the sorting signal within GLP-1 is masked when contained within the MPGF sequence. These results indicate that the sorting of proglucagon into secretory granules occurs prior to the initial processing event, such that processing occurs exclusively within the granule. Another possibility is that processing to glicentin and MPGF occurs first, with the prediction that glicentin is sorted into granules and processed to glucagon, while MPGF is not sorted, or very inefficiently sorted into granules. Given the emerging evidence of significant amounts of GLP-1 being produced in alpha cells in healthy human islets (37), this model would predict that proglucagon is first sorted into granules, then processed by both PC2 and PC1/3 to yield glucagon and GLP-1, respectively. This proglucagon processing profile changes in diabetes; in human and rodent islets, there is a significant increase in the processing of proglucagon to GLP-1. This alteration in the processing profile was first shown in rats treated with STZ; both PC1/3 and PC2 mRNA levels increased, concomitant with increases in glucagon and GLP-1 secretion (38). These results have since been corroborated in several studies showing significant elevations in PC1/3 expression and activity and GLP-1 production and secretion in αTC1-6 cells (39) and InR1G9 cells (40) grown in media containing high glucose concentrations, well as in islets from rodent models of diabetes. A recent study showed that up to 40% of alpha cells in human islets obtained from healthy donors contain both GLP-1 and glucagon and this proportion increases to 60% in islets from donors with Type 2 diabetes (37). The expression of PC1/3 and the production of GLP-1 and glucagon are increased in response to the diabetes-related pro-inflammatory cytokine, IL-6, in isolated human and mouse islets (41), and in the db/db (42), and ob/ob (43) mouse models of obesity-associated diabetes. Alpha TC1 cells engineered to be depleted in PC2 overexpress PC1/3, and subsequently produce sufficient amounts of GLP-1 to overcome STZ-induced hyperglycemia in mice (44, 45). In contrast to these findings, islets from the NOD mouse model of type 1 diabetes mouse showed a small but significant increase in GLP-1 and PC1/3 in alpha cells (42), but no change in the amount of secreted intra-islet GLP-1.

If both glucagon and GLP-1 are produced in a proportion of alpha cells, and are both sorted to secretory granules, the question arises: are they sorted to distinct granule populations, and released under different glucose conditions? These questions may have been answered in a very recent islet granule peptidomics study showing that both human and mouse islets produce 300-1000 times more glucagon than active GLP-1 (46), indicating that the processing of proglucagon to active GLP-1 in alpha cells is very inefficient. Also in this study, analysis of secreted proglucagon-derived peptides showed that both glucagon and active GLP-1 were released in parallel in response to either low (1 mM), medium (6 mM) or high (16.7mM) glucose concentrations. Therefore, both glucagon and GLP-1 are likely stored in the same granules and secreted under the same conditions, with glucagon being the dominant peptide, and perhaps serving as the intra-islet GLP-1R agonist (47).

## Clues to Hyperglucagonemia Lie in Alpha Cell Biology

The control of hyperglucagonemia obviously targets glucagon secretion. But what mechanism(s) are potentially druggable? Inhibition of glucagon secretion by glucose from alpha cells is a long-standing puzzle in islet biology. Unlike insulin secretion from beta cells which is primarily driven by prevailing glucose levels, there is no one single factor that governs glucagon secretion from the alpha cell. Intrinsic glucose sensing, intra-islet paracrine secretion and factors from the alpha cell itself all interact to generate a complex network that regulates glucagon secretion.

### Direct Effects of Glucose on the Alpha Cell

**Figure 2:**
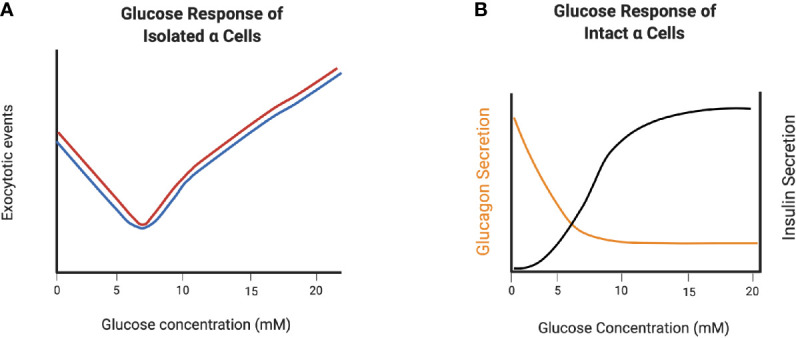
Glucagon secretion from dispersed alpha cells and alpha cells in intact islets demonstrate the role of paracrine regulation at high glucose concentrations. **(A)** V-shape curve of glucagon exocytosis in response to glucose in dispersed non-diabetic (black) and T2D (red) human α-cells. **(B)** Glucagon secretion from intact islets in response to glucose. Created with BioRender.com.

In order to examine the direct effects of glucose on glucagon secretion in the absence of paracrine inputs, isolated mouse pancreatic alpha cells, clonal hamster In-R1-G9 cells (48, 49), clonal mouse αTC1-6 and -9 cells (39, 50, 51) and dispersed alpha cells from human islets (52) have been used. All of these preparations show a bimodal response to increasing glucose concentrations. In the range from 1 to ~7 mM, glucagon secretion is suppressed in a dose-dependent manner, and above 7 mM, glucagon secretion increases (**Figure 2A**). This secretion profile suggests intrinsic mechanisms alone can operate in regulating glucagon secretion below 7 mM glucose, and that these mechanisms may be ineffective at higher glucose concentrations. However, such conclusions must be interpreted with caution, as single dispersed alpha cells are in a highly abnormal environment, and alpha cell lines are not representative of the normal alpha cell phenotype, as discussed in more detail below.

The alpha cell secretory response to both glucose is likely more accurately captured in isolated, intact mouse and human islets, where the paracrine regulatory environment and cell-cell contacts are intact. Similar to dispersed alpha cells, increasing the glucose concentration from 1 to 7 mM dose dependently decreases glucagon secretion from mouse alpha cells (53) and human alpha cells (52) within intact islets, and remains low as glucose levels increase beyond 7 mM, a concentration at which insulin secretion is stimulated (**Figure 2B**). Therefore, paracrine inputs are significant factors in the inhibition of glucagon secretion as glucose concentrations increase above euglycemia.

One mechanism underlying the intrinsic response to glucose is the direct effect on alpha cell electrical activity. At low (1 mM) glucose concentrations, alpha cells in intact mouse and human islets exhibit low K_ATP_ activity and are electrically active (54–56) and as glucose concentrations increase, K_ATP_ activity is inhibited. This is an apparent physiological paradox, since further closure of K_ATP_ channels should more strongly depolarize the plasma membrane and result in opening of voltage-dependent Ca^2+^ channels (VDCCs), as in beta cells. In alpha cells, glucagon exocytosis is coupled to the opening of P/Q-type VDCCs (55). A recent review by Zhang et al. (57) nicely explains this apparent paradox: membrane depolarization evoked by K_ATP_ inhibition reduces the amplitude of action potentials from Na^+^ channels. It is this reduction in the amplitude of action potentials that result in the closure of the voltage-dependent P/Q-type Ca^2+^ channels, leading to inhibition of glucagon granule exocytosis. Therefore, the intrinsic regulation of glucagon secretion by glucose may be explained primarily by the unique electrical properties of the alpha cell, and secondarily by glucose metabolism.

It has been also proposed that another mechanism for the direct effect of glucose on glucagon secretion is the regulation of intracellular Ca^2+^ flux through store-operated current (SOC) (58, 59). Under low glucose conditions, Ca^2+^ depletion of the ER causes the translocation of stromal interaction molecule 1 (STIM1) from the ER to the subplasmalemmal junctions, leading to clustering with Orai1, resulting in SOC activation. In contrast, exposure of alpha cells to high glucose conditions and subsequent high ATP levels activates the sarcoendoplasmic reticulum calcium transport ATPase (SERCA) pump, increasing Ca^2+^ sequestration from the cytoplasm into the ER. Filling the store with Ca^2+^ inhibits the translocation of STIM1, turns off CRAC channel subunit, reduces VDCC activity and suppresses glucagon secretion (59, 60). However, this hypothesis implicated L-type VDCCs in glucagon exocytosis, which have been shown to mediate intracellular Ca^2+^ oscillations, but not granule exocytosis, in alpha cells (54).

There is evidence that glucagon secretion becomes uncoupled from intracellular Ca^2+^ levels under high glucose conditions (61), suggesting the presence of alternate or additional intracellular signalling pathways in the regulation of glucagon secretion. In particular, cAMP signalling may play a key role in the alpha cell secretory response to insulin and somatostatin (62). There is one report that cAMP may also mediate intrinsic glucose sensing within the alpha cell. Using genetically encoded fluorescent cAMP biosensors, it was shown that high glucose suppressed subplasmalemmal cAMP levels in isolated mouse and human islets (63). This effect was not abolished in Ca^2+^-free media, nor in the presence of insulin receptor or SSTR2 antagonists. Conversely, sustained high cAMP levels abolished the suppression of glucagon secretion by high glucose concentrations. There may specific isoforms of adenylyl cyclase and phosphodiesterases that are associated with cAMP-regulated glucagon secretion/hyperglycemia [reviewed in (64)], and exactly how glucose interacts with these components of cAMP signalling remains to be determined.

Lastly, intrinsic glucose sensing by the alpha cell may also be mediated by the nutrient sensors AMP-activated protein kinase (AMPK) and its downstream target, mammalian target of rapamycin complex 1 (mTORC1). In a series of studies that manipulated alpha cell expression of AMPK itself (65) and its upstream effectors PASK (66) and LKB1 (67), it was shown that components of this nutrient-sensing pathway can mediate the low glucose-induced secretion of glucagon. One of these proteins, PASK, is down-regulated in T2D human islets, thus indicating that components of the AMPK pathway may be potential targets for controlling hyperglucagonemia. Using innovative mouse models that selectively targeted activators and inhibitors of mTORC1, it was shown that loss of mTORC1 activity resulted in a loss of the glucose counter-regulatory response and reduction in response to alpha cell secretagogues (68). Interestingly, depletion of the mTORC1 inhibitor TSC2 in alpha cells resulted in a mouse model of hyperglucagonemia and glucagon resistance (69), which will be an excellent resource for studies on mechanisms of hyperglucagonemia.

Therefore, the mechanisms underlying the intrinsic response to glucose may provide potential targets for the control of abnormally up-regulated glucagon secretion in diabetes.

## Intra-Islet Regulation of Glucagon Secretion: Changes in the Alpha Cell Response to Secreted Factors From the Beta Cell

The beta cell secretory granule contains a number of agents that act directly or indirectly on the alpha cell to inhibit glucagon secretion, and also generally modulate mechanisms of alpha cell biology, such as proliferation. Insulin, the primary cargo, is a potent suppressor of glucagon secretion and operates through several mechanisms. Upon binding to its receptor on the alpha cell, signaling through PI3 kinase is activated, reducing K_ATP_-channel activity, causing plasma membrane hyperpolarization and reduced activity of the P/Q type Ca^2+^ channel (70). Mice lacking the insulin receptor on alpha cells (αIRKO) exhibit hyperglycemia and hyperglucagonemia, indicating that insulin receptor signalling is required for an appropriate alpha cell secretory response to glucose (71). Alpha cell insulin resistance may underlie the abnormal up-regulation of glucagon secretion Type 2 diabetes (72). Additionally, these results also indicate that insulin alone is not sufficient to regulate glycemia in the face of hyperglucagonemia.

Along with insulin, gamma amino butyric acid **(**GABA) is also released from the beta cell and is a potent suppressor of glucagon secretion from alpha cells (73, 74). Activating the GABA_A_ receptor in alpha cells results in Cl^-^ influx into the cells which hyperpolarizes the membrane and reduces glucagon secretion (75). As well, there is coordination between insulin and GABA_A_ receptor activity, as insulin action leads to the translocation of GABA_A_ receptor to the cell membrane (76), thus augmenting the inhibitory effects of GABA. In addition, GABA also inhibits mTOR activity to suppress alpha cell proliferation. In type 1 diabetes, the destruction of beta cells leads to a reduction in the amount of secreted GABA, resulting in the activation of mTOR and alpha cell proliferation (77). In addition to effects on alpha cell proliferation, some studies have suggested that pharmacologic activation of GABA_A_ receptor by artemisinins or GABA may alter alpha cell identity and trans-differentiate adult alpha cells to beta-like cells (78–80), and have led to clinical trials investigating GABA receptor agonists as protection against the development of diabetes. However, there is still some debate on this topic, as transdifferentiation could not be induced either in isolated mouse islets in which both insulin and glucagon were tagged with fluorescent reporters (81) or in an alpha cell-specific lineage tracing model (82). In any case, the reported immunomodulatory effects of GABA, together with either GLP-1 (83) or the SGLT2 inhibitor empagliflozin (84) also protect newly formed beta cells in the inflammatory environment of T1D, and thus also indirectly restore normal regulation of alpha cell mass and glucagon secretion.

Other beta cell secreted factors that have direct effects on the alpha cell include serotonin, adenosine, and Zn^2+^. Direct effects of serotonin are mediated by activation of the serotonin receptor, 5-HT_1F_R, on α-cells, which reduces intracellular cAMP to suppress glucagon secretion (85, 86). In patients with long-standing T2D, the proportion of alpha cells expressing 5-HT_1F_R is decreased, suggesting that reduced serotonin action on alpha cells may play a role in hyperglucagonemia of diabetes. In STZ-treated mice, administration of the 5-HT_1F_R agonist LY344864 alleviated hyperglucagonemia and hyperglycemia. However, insulin-induced hypoglycemia was worsened, suggesting that the effects of serotonin are glucose-independent (85). Therefore, while alpha cell HT_1F_R may be a potential target for the treatment of hyperglucagonemia, it may not be an ideal target.

The effects of adenosine are mediated by the adenosine A1 receptor (Adora1), in which activation is coupled to opening of K_ATP_ channels, hyperpolarization of the cell membrane and prevention of granule exocytosis. In NOD mice, autoantibody-positive people and people with long-term T1D, alpha cells gradually lose Adora1 expression, suggesting that the hyperglucagonemia of diabetes is associated with a loss of adenosine action (87).

The Zn^2+^ transporter, ZnT8, is encoded by *Slc30A8*, and variants of this gene are associated with an increased risk of T2D and impaired glucose tolerance. ZnT8 is located in the secretory granule membrane of both α-and β-cells. There is a direct relationship between expression of the proglucagon gene and *Slc30A8* in α-cells (88). However, there are conflicting findings about the effects of Zn^2+^ on glucagon secretion (88). There are reports that, in isolated mouse islets and alpha cells, Zn^2+^ administration decreases glucagon secretion (89), or has no effect (90). In contrast, treatment of isolated human islets with similar concentrations of Zn^2+^ enhanced glucagon secretion (55). The reason for these discordant results is not clear; it may be that exogenously administered Zn^2+^ or Zn^2+^ secreted from the beta cell does not have a direct role in glucagon secretion in normal physiology. Interestingly, in mice lacking *Slc30A8/*ZnT8 specifically in alpha cells, there is a heightened secretory response to 1 mM glucose (91), and overexpression of ZnT8 specifically in alpha cells restricted glucagon secretion in response to 1 mM glucose (92), suggesting autocrine regulation by Zn^2+^.

## How Does Delta Cell Activity Inhibit Glucagon Secretion?

Somatostatin is a well-known tonic inhibitor of glucagon secretion. Somatostatin binds to the SSTR2 receptor subtype on alpha cells (93), which is coupled to the inhibitory G_i_ subunit, resulting in decreased production of cAMP as a mechanism for the suppression of glucagon secretion (62). Another mechanism may be more closely coupled to Ca^2+^ flux and inhibition of glucagon granule exocytosis by de-priming of the alpha cell secretory granules located near L-type Ca^2+^ channels (94). Notably, secretion of somatostatin and inhibition of glucagon secretion both occur at 3 mM glucose, indicating that the alpha cell response to low glucose may be fine-tuned by somatostatin (95). In rat pancreatic preparations perfused with an SSTR2 antagonist, the suppression of glucagon secretion by 3.5-5 mM glucose was lost (96), suggesting that somatostatin may be required for the fine control of glucagon secretion at euglycemia. However, in isolated human islets, blockade of SSTR2 did not affect suppression of glucagon secretion at 6 mM glucose (55), perhaps reflecting species-specific differences or differences in the models (perfused pancreas *vs* static islet culture).

There is also evidence that somatostatin is required for the high glucose-induced suppression of glucagon secretion: in perifused islets from mice lacking the prosomatostatin gene (*SST^-/-^* mice) (97), glucagon secretion was elevated at 10 and 20 mM glucose. Interestingly, insulin secretion was also elevated, indicating that both insulin and somatostatin are required for the suppression of glucagon secretion at high glucose concentrations. In intact human islets, high glucose (10 mM) inhibition of glucagon exocytosis was lost after administration of the SSTR2 antagonist CYN154806 (52). In diabetes, circulating and pancreatic somatostatin, together with SST mRNA, are elevated. However, expression of SSTR2 on alpha cells is decreased in T2D due to increased receptor internalization (52), indicating alpha cell somatostatin resistance. Together with alpha cell insulin resistance, this could be another mechanism in the hyperglucagonemia of diabetes. Alternatively, somatostatin resistance may be a dominant and direct mechanism of hyperglucagonemia, as eliminating the insulin receptor on delta cells completely abolishes the glucagonostatic effect of insulin, indicating an indirect glucagonostatic effect for insulin (98).

The emerging role of somatostatin in the regulation of alpha cell function and glucagon secretion has been further highlighted by one study in which mice were engineered for optogenetic activation of beta cells to study the paracrine regulation of alpha cells (99). By this approach, opto-activation of beta cells both suppressed alpha cell electrical activity and stimulated action potentials in delta cells mediated by gap junction currents. The suppressive effect of beta cell activation was lost in the presence of the SSTR2 antagonist CYN 154806 (99), indicating that somatostatin secretion stimulated by beta cell electrical activity is critical for the suppression of glucagon secretion. Subsequent modelling predicted that a reduction in gap junction connections between beta and delta cells, perhaps caused by disruptions in islet architecture in T2D (100), may contribute to the hyperglucagonemia of diabetes. Thus these findings highlight a central role for delta cells in the context of intra-islet regulation of glucagon secretion, and may have implications for designing drugs for the treatment of hyperglucagonemia of diabetes.

## The Alpha Cell as a Regulator of Glucagon Secretion: Known and Emerging Pathways

The alpha cell itself displays plasticity during the progression of diabetes. In addition to the mechanisms above that describe changes in responses to glucose and paracrine effectors, there are alterations within the alpha cell, including proglucagon processing and secretion of proglucagon-derived peptides, and remodelling of the secretory granules themselves in terms of exocytotic behavior and contents, and alterations in intracellular trafficking pathways.

Secreted glucagon from alpha cells can stimulate its secretion through an autocrine effect. It has been shown that glucagon stimulates glucagon secretion from the rat and mouse isolated alpha cells in an autocrine manner through glucagon receptor-stimulated cAMP signaling (101). In αTC1-9 cells and mouse islets, exogenous glucagon administration, as well as secreted glucagon stimulated by 1 mM glucose, increased glucagon secretion and proglucagon gene transcription through the PKA-cAMP-CREB signalling pathway in a glucagon receptor-dependent manner (102). The apparent interplay between glucagon and its receptor on the alpha cell appears to be of a positive feedback loop, controlled by the pulsatile nature of glucagon secretion.

In addition to glucagon, a novel proglucagon-derived peptide, proglucagon 1-61 (PG 1-61) comprised of GRPP and glucagon, was identified as a major molecular form of glucagon in plasma from human patients with hyperglucagonemia-associated conditions: Type 2 diabetes and renal dysfunction, morbid obesity or gastric bypass surgery, and only after oral ingestion of macronutrients (103). This N-terminally extended form of immunoreactive glucagon was not found in healthy controls, leading the authors to speculate that PG 1-61, and molecular heterogeneity of glucagon in general, could be a biomarker for alpha cell dysfunction. Administration of PG 1-61 decreased glucagon secretion in healthy rats, diverging from the positive feedback observed with glucagon administration. Interestingly, this effect was not observed in diabetic rats, suggesting an impairment in this distinct feedback loop in the alpha cell.

**Figure 3:**
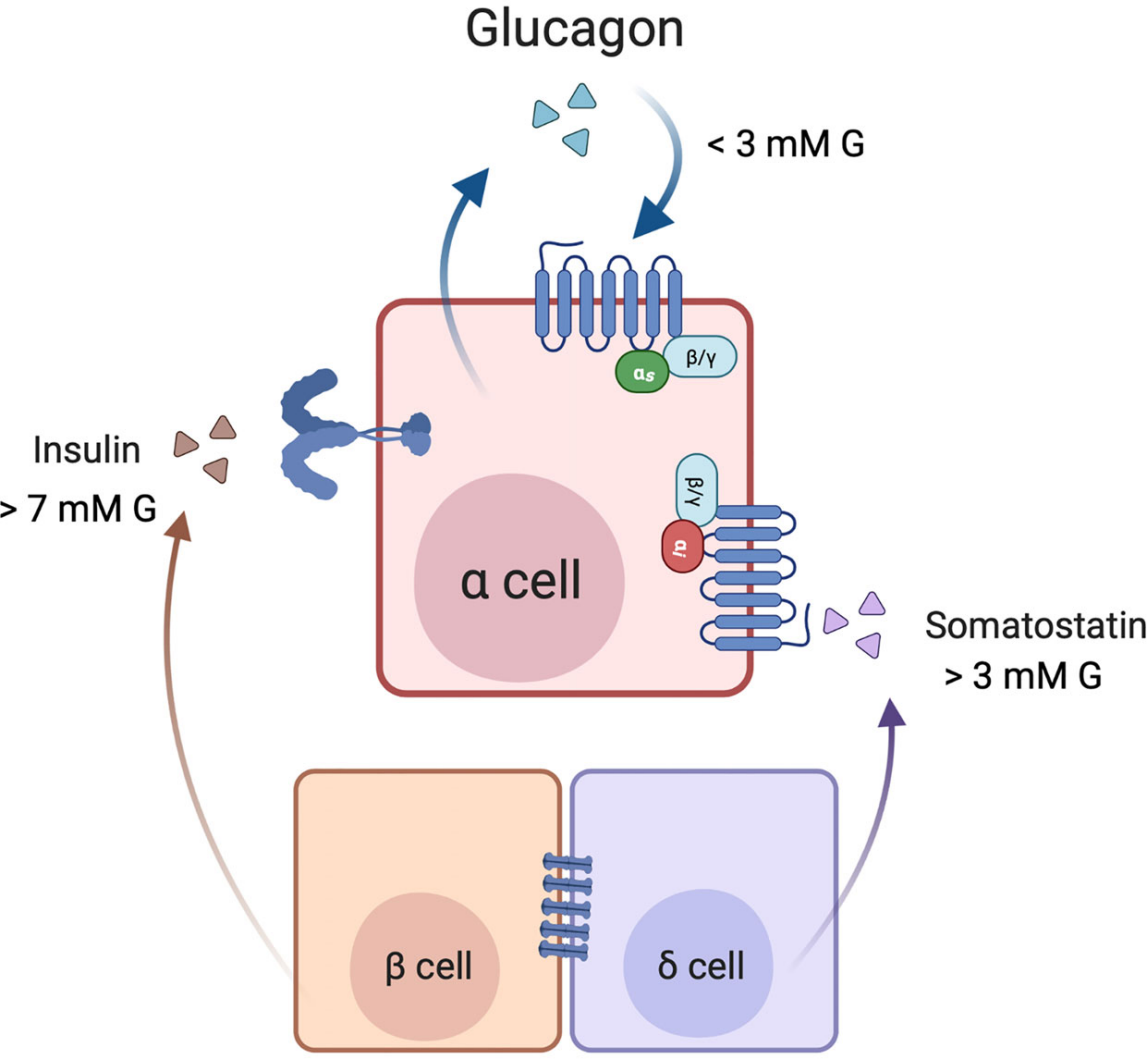
Cross-talk among α, β, and δ-cells in the paracrine regulation of glucagon secretion. Under low glucose (1-3 mM) conditions, secreted glucagon may act in an autocrine feed-forward loop. In high glucose (> 7 mM) conditions, glucose metabolism in β cells leads to insulin release, and insulin inhibits glucagon secretion by signalling through its receptor on the alpha cell. Additionally, electrical coupling of the beta and delta cells through gap junctions contributes to somatostatin release. Somatostatin may also be secreted independently of insulin when glucose concentrations are > 3 mM. Somatostatin binds to SST receptor 2 (SSTR2) on the α cell membrane, where signalling through G_i_ inhibits glucagon secretion. Created with BioRender.com.

The interplay between glucagon, insulin and somatostatin in the regulation of glucagon secretion at various levels of glucose is illustrated in **Figure 3**. In diabetes, beta cell deficiency, together with alpha cell insulin and somatostatin resistance, all contribute to alpha cell dysfunction and a loss of the regulation of glucagon secretion, resulting in hyperglucagonemia.

## Role of GLP-1: Intra-Islet or Intestinal?

The glucose-dependent insulinotropic actions of intestinal GLP-1 on the beta cell are well known. GLP-1 also suppresses glucagon secretion in both healthy people and people with type 2 diabetes (104), and poorly-controlled type 1 diabetes (105). The emerging evidence of GLP-1 being produced and secreted by the pancreatic alpha cell has led to a debate on which source of GLP-1 suppresses glucagon secretion from pancreatic alpha cells. To investigate this question, Chambers et al. (106) generated a *Gcg* knockout mouse and then by reactivation of *Gcg* in L-cells or alpha cells, showed that islet-generated GLP-1 was primarily responsible for glucose homeostasis by promoting glucose-stimulated insulin secretion and suppressing glucagon secretion. The gut-derived GLP-1 binds to its receptor on local afferent vagal nerve terminals, which ultimately signals for satiety, delaying gastric emptying and suppression of hepatic glucose release (106, 107). However, this model may not translate well to human islets due to differences in islet architecture, and in light of the recent findings that glucagon is the dominant peptide hormone secreted from human alpha cells (46).

The search for a GLP-1 receptor on alpha cells has been hampered by a lack of a reliable GLP-1 receptor antibody (108, 109). GLP-1 appears to mildly reduce action potentials in the alpha cell membrane at 1 mM glucose in isolated mouse alpha cells, and this effect is blocked by the GLP-1R antagonist exendin (9-39) (110), therefore suggesting the presence of GLP-1R, perhaps at a very low density, on a small proportion of alpha cells. The development of near infra-red and fluorescent analogues of GLP-1R ligands has enabled both *in vivo* (111, 112) and high-resolution tissue imaging (113, 114) of GLP-1R with high specificity, sensitivity, and reproducibility. Coupled with super-resolution microscopy, the fluorescent analog LUXendin645 revealed that 5% of alpha cells in murine islets expressed GLP-1R (114). Given the already small proportion of alpha cells in the mouse islet, the contribution of direct alpha cell action to the glucagonostatic effect of GLP-1 is likely very small.

Islet GLP-1 may also exert its effects through receptors on delta cells (115), resulting in stimulation of somatostatin secretion and inhibition of glucagon secretion *via* SSTR2 on alpha cells (116, 117). This paracrine effect could not be detected in isolated normal human islets (110); nonetheless, this mechanism may be clinically relevant in the treatment of T2D, as experiments in human islets showed that the GLP-1R agonist liraglutide enhanced somatostatin secretion to reduce hyperglucagonemia induced by the SGLT2 inhibitor dapagliflozin (118).

## Secretory Granule Dynamics and Hyperglucagonemia

As drugs targeted to the control of glucagon secretion are now being developed for the treatment of hyperglucagonemia, a deeper understanding of the dynamics of the alpha cell secretory granule is critical for identifying effective targets. However, the study of glucagon granule trafficking and exocytosis presents several technological challenges. Commonly used cell lines such as InR1-G9, αTC1-6 and αTC1-9, while useful for preliminary studies on trafficking and secretion, as a rule do not exhibit robust secretory responses to glucose or other secretagogues. The αTC1-6 cell line in particular differs from primary alpha cells in their complement of transcriptional, epigenetic and metabolic factors (119, 120) which may explain the blunted secretory response to glucose. Dispersed primary alpha cells may offer a slightly better alternative, but as discussed above, both cell lines and dispersed primary alpha cells exhibit aberrant glucagon exocytosis patterns at high glucose levels, likely due to the absence of paracrine inputs and juxtamembrane contacts. The greatest advances in gleaning the mechanisms of glucagon granule exocytosis have been made using patch-clamp approaches in isolated rodent or human islets. In such preparations, alpha cells can identified by their unique electrophysiological signature under low glucose conditions (121) or, in the case of mouse islets, by genetically-encoded fluorescence reporters such as YFP (122, 123) or tdTomato (124).

After proglucagon processing and granule maturation, glucagon is stored in the alpha cell secretory granule until a stimulus triggers exocytosis. As in beta cells, there may be different functional pools of secretory granules: a reserve pool and a readily releasable pool that is primed and situated at the sites of exocytosis. Quantitative ultrastructural analysis of murine islets has shown that, in the presence of 1mM glucose, the mouse α-cell contains ~4400 secretory granules, of which ~140 are in close proximity to the plasma membrane, or primed (125). This means that the reserve pool is large and can resupply the readily releasable pool to maintain euglycemia over extended periods of time. In the presence of 16.7 mM glucose, the numbers of the docked secretory granules increase to ~310, due to both decreased secretion and increased docking in response to increased glucose metabolism (125). Kinetic modelling of the exocytotic behaviour of alpha cell secretory granules under low glucose conditions predicts the relationship between intracellular Ca^2+^ levels and granule dynamics and mobilization, by which sequential Ca^2+^ binding events promote granule fusion with the plasma membrane (126).

Following docking, secretory granules are primed through the action of the SNARE protein complex. This complex contains two subsets of proteins; *i)* the t-SNAREs syntaxin 1A and SNAP-25, located in the plasma membrane; and *ii)* the v-SNAREs VAMP2 and synaptotagmin VII, which are located in the granule membrane (127). The Ca^2+^ dependence of glucagon granule exocytosis is due to the actions of synaptotagmin VII (128), a major Ca^2+^-responsive component. Under low glucose conditions, SNAP-25 and syntaxin 1A are translocated to the plasma membrane. SNAP-25 itself may play a role in the transportation of granules from the releasable pool to the readily releasable pool, and then mediates their fusion with plasma membrane *via* interaction with syntaxin 1A (125, 126). Following Ca^2+^ influx and binding, the C2 domain of synaptotagmin VII binds to syntaxin 1A and forms the SNARE complex to prime the secretory granules for exocytosis (125) in a mechanism similar to that in beta cells (129, 130).

Alpha cell membrane SNAREs are located in close proximity to some of the ion channels that govern alpha cell membrane depolarization and Ca^2+^ entry, possibly at the site of cholesterol-rich lipid microdomains (131). Modelling data predicts that membrane microdomains of P/Q-type Ca^2+^ channels are closely coupled to glucagon exocytosis (132), suggesting that granules in the readily releasable pool cluster at specific sites within the membrane. Live imaging of exocytosis using a proglucagon-luciferase reporter showed spatial clustering of glucagon secretion sites in αTC1-6 cells (133). Future studies may reveal some interesting dynamics with SNARE proteins that may fine-tune the alpha cell secretory response to glucose and paracrine inputs.

Could disruption of these molecular mechanisms contribute to the hyperglucagonemia of diabetes? In dispersed alpha cells from patients with T2D, voltage-dependent Ca^2+^ currents were normal; nonetheless, secretory granules remained in the reserve pool for a longer period of time compared to non-diabetic alpha cells, and exocytosis was actually impaired (52). However, neither membrane potential nor exocytosis was responsive to insulin or (to a greater extent) somatostatin, in contrast to normal alpha cells in which both were significantly reduced. Therefore, in T2D, hyperglucagonemia may result from insulin and somatostatin resistance at the level of the readily releasable pool of granules. In alpha cells of patients with T1D, expression levels of genes encoding SNARE proteins, ion channels and cAMP signalling molecules were disrupted (134), which could explain the impaired glucose counter-regulatory response and the inappropriately elevated levels of postprandial glucagon in T1D. Combining patch-clamp electrophysiological measurements with single-cell RNA sequencing (patch-seq) in human islets has given high-resolution insight into mechanisms underlying impairments in alpha cell function in diabetes (135) at the level of granule exocytosis. Further characterization of the link between electrophysiological signatures and the genes regulating the dynamics of granule exocytosis will reveal new mechanisms of alpha cell dysfunction in diabetes.

## Novel Proteins in the Regulation of Glucagon Trafficking and Secretion

Identifying new pathways or networks that control glucagon granule biogenesis and trafficking may identify novel targets for the control of hyperglucagonemia in addition to yielding a greater understanding of alpha cell biology in both health and disease. There is an emerging hypothesis that glucagon secretion can be controlled by trafficking through the endosomal-lysosomal pathway, similar to insulin (136), and below, we highlight some recent studies that suggest glucagon may regulated through such an alternate trafficking pathway.

Brefeldin A-inhibited guanine nucleotide exchange protein 3 (BIG3) is a member of the Arf-GEF family of proteins, and was initially found in a database search and found to inhibit insulin granule biogenesis and insulin secretion (137). A subsequent study found that it had a similar role in regulating glucagon granule production and exocytosis (138). Although these studies inferred action of BIG3 through whole-body knockouts and not cell-specific or conditional knockouts, some more recent studies have shown that BIG3 is localized to LAMP1^+^ lysosomes in hypothalamic neurons (139), thus implicating BIG3 in lysosomal trafficking, at least in neurons. Whether BIG3 can mediate glucagon trafficking through lysosomes remains to be investigated.

**Figure 4:**
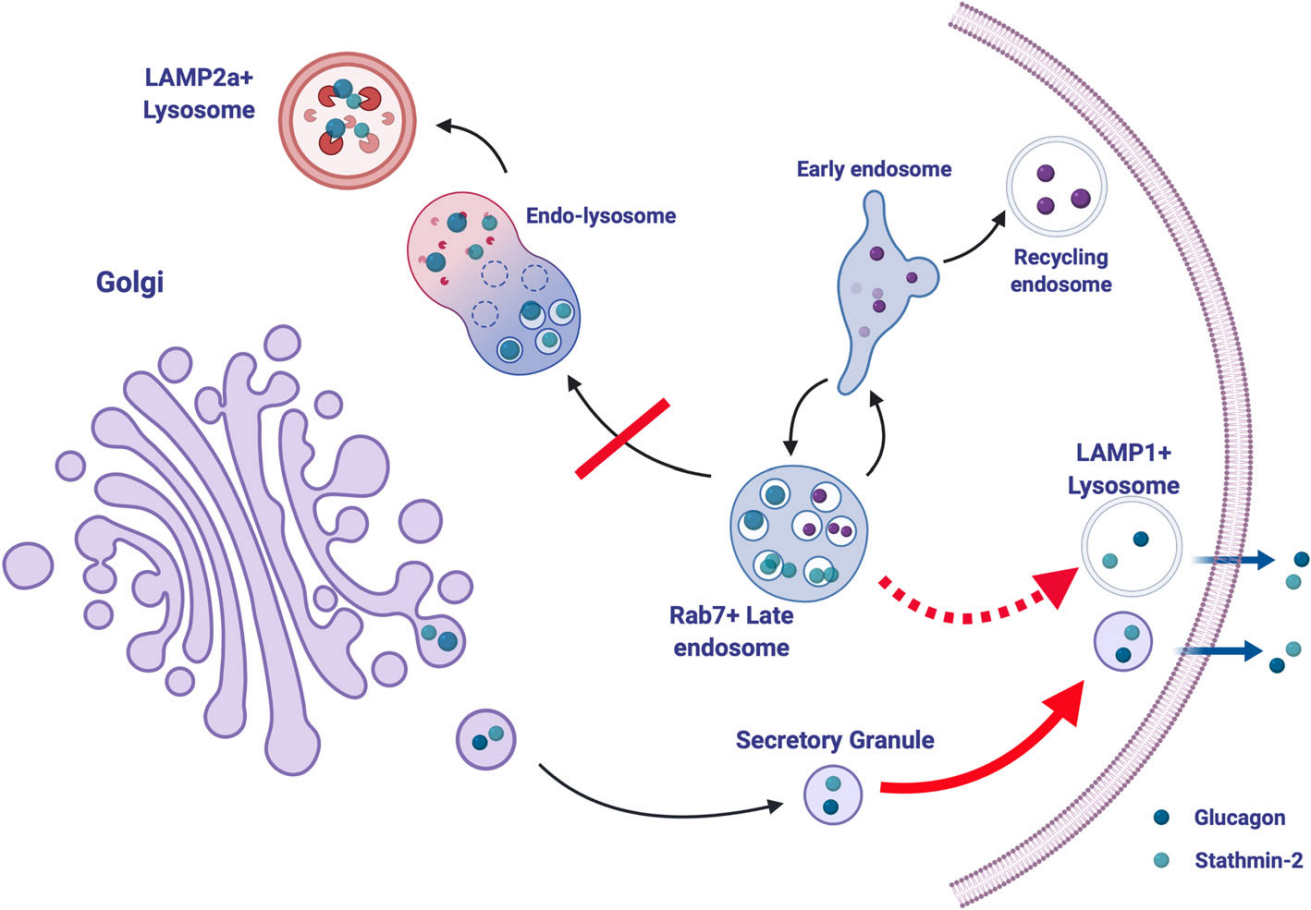
Stathmin-2-mediated lysosomal trafficking modulates glucagon secretion. Glucagon (dark blue) and stathmin-2 (light blue) are normally sorted to secretory granules from the Golgi in alpha cells. Stathmin-2 overexpression diverts glucagon-containing secretory granules to lysosomes (black arrows), thus reducing glucagon secretion. In the relative absence of stathmin-2, this regulatory pathway is disrupted; transport of glucagon and stathmin-2 to the LAMP2^+^ lysosome is inhibited and instead is re-routed to lysosomes (possibly LAMP1^+^) adjacent to the plasma membrane, thus and allowing for excess glucagon secretion (dotted red arrow). Additionally, secretion from secretory granules is also enhanced (solid red arrow). Created with BioRender.com.

The composition and cargo of the alpha cell secretory granule may also hold some determinants of glucagon secretion. While it is known that granule contents and composition are modified during normal granule maturation, a more complete picture of granule remodeling and heterogeneity in the context of intracellular trafficking networks in normal physiology and in diabetes is required. In an effort to identify networks of secretory granule proteins that interact with glucagon and regulate its trafficking and secretion, proteomic analysis was conducted on αTC1-6 cell secretory granule lysates immunoprecipitated with tagged glucagon (51). This qualitative study demonstrated the plasticity in the network of proteins interacting with glucagon in response to insulin or GABA under high (25 mM) or low (5.5 mM) glucose. Stathmin-2, a member of the family of neuronal phosphoproteins that associates with the secretory pathway in neurons, was identified as a candidate protein for the regulation of glucagon secretion and subsequently shown to modulate glucagon secretion through the lysosomal pathway (140) and may be down-regulated in diabetes in humans (141) and in mice (142). Therefore, disruptions in the routing of glucagon through the lysosomal pathway may contribute to the hyperglucagonemia of diabetes (**Figure 4**).

Glucagon trafficking and exocytosis may also be controlled through nutrient-driven pathways. The nutrient sensor O-GlcNAc transferase (OGT) catalyses the O-glycosylation of several proteins including those involved in the conventional secretory pathway (143) and autophagosome-lysosome fusion (144). In mice lacking OGT specifically in alpha cells, glucagon secretion, cell content and alpha cell mass are reduced (145). Possible mechanisms include lack of O-glycosylation of FOXA1 and FOXA2, which regulate genes encoding proteins involved in proglucagon processing and glucagon secretion (146). Whether other trafficking proteins are affected, and how alpha cell function is affected in diabetes in these mice, is not yet known.

So what are the implications of glucagon trafficking through the lysosomal pathway in diabetes? Lysosomal trafficking and autophagy in the beta cell may be a possible mechanism of insulin secretory defects in diabetes, with a recent study providing evidence for impairment of lysosomal function in human T1D (147). How does lysosomal function contribute to defects in alpha cell function? It is tempting to hypothesize that impairments in lysosomal biogenesis and trafficking result in both reduced insulin secretion in the beta cell and unregulated glucagon secretion from the alpha cell. Further investigation into the altered dynamics of glucagon trafficking in the alpha cell in diabetes may reveal key roles for the lysosome in the regulation of glucagon secretion, thus identifying a potential new target for the treatment of hyperglucagonemia.

## Advanced Tools for the Study of Alpha Cell Biology in Diabetes

Finally, some excellent single-cell transcriptomics and epigenomics databases are being generated that reveal the dynamics of intracellular trafficking networks at the transcriptional level in human pancreatic alpha cells in both health and diabetes (148–150). The mapping of T2D-associated genetic variants with RNA-seq of human islets (151) may reveal risk factors associated with defects in alpha cell function A novel immunocompromised mouse model in which glucagon-encoding codons were deleted while preserving both GLP-1 and GLP-2 will provide an innovative and much-needed resource for the study of the regulation of glucagon secretion from human islets *in vivo* (152). In this study, transplantation of islets from people with T2D resulted in hyperglucagonemia with apparent alpha cell insulin resistance, revealing intrinsic alpha cell defects in T2D. Moreover, defects in alpha cell function were more apparent than in isolated islets, thus emphasizing the utility of such an *in vivo* system to investigate the molecular mechanisms of glucagon secretion in human islets, and the testing of possible treatments for hyperglucagonemia.

## Concluding Thoughts on Targeting Glucagon Secretion for Treating Hyperglucagonemia of Diabetes

While the development of glucagon receptor antagonists and other inhibitors of glucagon action has provided some possibilities for the treatment of hyperglucagonemia, there are significant side effects that result from impaired hepatic metabolism and potentially uncontrolled alpha cell proliferation. The advantage to developing such drugs, however, lie in the fact that the glucagon receptor is an easily available target. In contrast, targeting glucagon secretion as a means to treat hyperglucagonemia may alleviate concerns about effects on the liver and alpha cell mass; however, there are potentially many more targets within the alpha cell secretory pathway, and many of those may not be easily accessible for drug treatment. The ongoing discovery of novel proteins and networks that regulate the secretion of glucagon will shed further light on alpha cell biology in health and disease while also searching for improved means to control hyperglucagonemia and hyperglycemia of diabetes.

## Author Contributions

SD and FA co-wrote the manuscript. FA was SD’s PhD student and this review was largely adapted from FA’s thesis. All authors contributed to the article and approved the submitted version.

## Funding

This work was funded by a Natural Sciences and Engineering Research Council Discovery Grant to SD.

## Conflict of Interest

The authors declare that the research was conducted in the absence of any commercial or financial relationships that could be construed as a potential conflict of interest.

## Publisher’s Note

All claims expressed in this article are solely those of the authors and do not necessarily represent those of their affiliated organizations, or those of the publisher, the editors and the reviewers. Any product that may be evaluated in this article, or claim that may be made by its manufacturer, is not guaranteed or endorsed by the publisher.
